# Retrospect of the Progress of Surgical Literature for the Year 1838-9

**Published:** 1839-10

**Authors:** 


ART. XVII.—*Retrospect of the Progress of Surgical Literature for the Year* 1838-9. By Messrs. NEWNHAM, W. WICKHAM, and SALTER. Read, June 1839, before the Annual Meeting of the Southern Branch of the *Provincial Medical Association,* and published at its request. —*London,* 1839, 8vo, pp. 44.

THIS is a very excellent report, comprehending in brief space notices of all the important additions made to surgical science during the past twelve months, and illustrating each by a judicious commentary. It cannot fail to be useful to every practitioner, and more particularly to those who have not access to the recent surgical literature of France. We quote the following observations on the subject of revaccination, as well because of their intrinsic importance, coming as they do from a gentleman of great experience and accurate observation, as because they constitute a critical commentary on the views of Professor Heim, first communicated to the profession in this country in the Thirteenth Number of this Journal.

"On the subject of revaccination, your reporters can only appeal to further experience; 'adhuc sub judice lis est.' But they would endeavour to bring their quota of observation towards the settlement of this great question. Professor Heim states the efficacy of revaccination to consist in the complete progress of the vesicle. The experience of your reporters would lead them to say that it never was complete except in the almost solitary instance, which would confirm rather than invalidate the rule. In 150 cases of revaccination which have been noted by one of your reporters, only one could be said to approximate towards a regular vaccine vesicle; and this reporter is disposed to consider revaccination in no other light than as a test of the constitutional influence of the former vaccination. In the instances which have come under his notice, the local action has been observed much earlier than it ought to be, and, in some cases, has subsided without further mischief; in others, a vesicle has been soon formed with an undefined, ragged border, surrounded from the first by an inflammatory blush, and always wanting the defined circular edge of the genuine vesicle; much too much local action has been produced; there has been almost always swelling of the axillary glands, which is unusual in a first vaccination, unless the lymph has been too aged; there has arisen a much higher and a much earlier state of constitutional irritation, originating and ceasing at very uncertain periods, governed by the local cause, and leaving a sore arm with protracted swelling, and a flattened, undefined crust, instead of the regular, raised, well-defined chocolate-coloured scab. We are well aware that the statistical report (as well as other observations) of the result of revaccination of the Prussian army would seem to militate against these conclusions, but we believe that if tried by the above tests, a very different result would be exhibited. According to the views of your reporters, it follows either that we have no test of what is a genuine vaccine vesicle, or that this is not the progress of such vesicle. If we adopt the former proposition, vaccination may be abandoned; if the latter, it is evident that the symptoms are the result of the local agencies of a poison introduced into the constitution, but, like other specific agents, capable of producing its regular influence only once during life. Yet, if this be true, the efficacy of revaccination cannot consist in renewing its protecting influence over the constitution, but simply in forming the test referred to. Your reporters must vehemently protest against the employment of lymph taken later than the ninth day, and their experience would lead them to give the meed of incalculable superiority to that taken on the eighth. To the want of attention to this most important rule, many failures may doubtless be attributed; and also the probable origin of that unfortunate hypothesis which represents the necessity of going back to the cow for a renewal of lymph, which had become deteriorated or effete by passing through so many subjects; an hypothesis so contrary to nature, to reason, to science, and to fact, that we can only be surprised at its endurance. Notwithstanding the conflicting testimonies of the past year, your reporters would be disposed to believe that a genuine vaccine vesicle may be accelerated or retarded without impairing the security of the patient, provided that its course be otherwise regular; and they would also believe that the efficacy of vaccination in protecting its recipients from the fatal consequences of smallpox casually introduced into the system, is much greater than that of previous smallpox itself, whether natural or inoculated." (pp. 8-10.)

